# Morphological and molecular assessment of apoptotic mechanisms in peripheral neuroblastic tumours

**DOI:** 10.1038/sj.bjc.6603212

**Published:** 2006-06-06

**Authors:** S Uccini, C Colarossi, S Scarpino, R Boldrini, P G Natali, M R Nicotra, F M Perla, O Mannarino, P Altavista, C Boglino, C A Cappelli, D Cozzi, A Donfrancesco, G Kokai, P D Losty, H P McDowell, C Dominici

**Affiliations:** 1 Department of Experimental Medicine & Pathology, La Sapienza University, Viale Regina Elena 324, Rome I-00161, Italy; 2 Division of Pathology, Bambino Gesù Children's Hospital, Rome, Italy; 3 Laboratory of Immunology, Regina Elena National Cancer Institute, Rome, Italy; 4 Institute of Molecular Biology and Pathology, CNR, Rome, Italy; 5 Department of Pediatrics, La Sapienza University, Viale Regina Elena 324, Rome I-00161, Italy; 6 Section of Toxicology and Biomedical Sciences, ENEA Research Center Casaccia, Rome, Italy; 7 Division of Surgery, Bambino Gesù Children's Hospital, Rome, Italy; 8 Division of Oncology, Bambino Gesù Children's Hospital, Rome, Italy; 9 Division of Pathology, RLC NHS Trust Alder Hey, Liverpool, UK; 10 Division of Surgery, RLC NHS Trust Alder Hey, Liverpool, UK; 11 Division of Oncology, RLC NHS Trust Alder Hey, Liverpool, UK; 12 Laboratory of Oncology, Bambino Gesù Children's Hospital, Rome, Italy; 13 Division of Child Health, School of Reproductive and Developmental Medicine, Liverpool University, Liverpool, UK

**Keywords:** neuroblastoma, mitosis–karyorrhexis index (MKI), apoptosis, p53, tumour necrosis factor-related apoptosis-inducing ligand (TRAIL), caspases

## Abstract

Multiple defects in apoptotic pathways have been described in peripheral neuroblastic tumours (NTs). Mitosis–karyorrhexis index (MKI) is a reliable morphological marker identifying favourable and unfavourable NTs. The extent to which apoptotic processes contribute to determine the clinical significance of MKI is still undefined. Apoptosis was investigated in a series of 110 peripheral NTs by comparing MKI to immunohistochemical and molecular apoptotic features. High MKI was found in 55 out of 110 NTs (50%) and was associated with advanced stage (*P*=0.007), neuroblastoma (NB) histological category (*P*=0.024), MYCN amplification (*P*<0.001), and poor outcome (*P*=0.011). Overall survival probability was 45% in patients with high MKI compared to 73% in patients with low MKI. In the same 110 NTs, the expression of Bcl-2, Bcl-X_L_, Bax and Mcl-1 was studied by immunohistochemistry, but no significant associations were found with clinicohistological features. Microarray analysis of apoptotic genes was performed in 40 out of 110 representative tumours. No significant association was found between the expression of apoptotic genes and MKI or clinicohistological features. Proliferative activity was assessed in 60 out of 110 representative tumours using Ki67 immunostaining, but no significant correlations with MKI or clinicobiological features were found. In NTs, the combination of apoptosis and proliferation as expressed by MKI is a significant prognostic parameter, although neither of them is *per se* indicative of the clinicobiological behaviour and outcome.

Peripheral neuroblastic tumours (NTs) are embryonal tumours of the sympathetic nervous system that originate during fetal or early postnatal life as result of developmental defects during the normal differentiation from neural crest-derived progenitor cells to mature sympathetic neuronal cells (Nakagawara and Ohira, 2004). Morphologically, peripheral NTs can range from undifferentiated, truly malignant neuroblastoma (NB), via ganglioneuroblastoma (GNB) to well differentiated benign ganglioneuroma (GN). Clinical behaviour of NBs and GNBs is very heterogeneous since they can often regress spontaneously in patients less than 1 year of age, while they tend to behave aggressively in those over this age (Evans *et al*, 1976; De Bernardi *et al*, 2003). Accordingly, among patients with NB or GNB, different risk categories – defined as high, intermediate and low risk – can be identified according to clinicobiological features (Brodeur, 2003). Accumulating evidence suggests that tumour regression is at least in part regulated by developmentally programmed neuronal cell death and/or differentiation that are similar to the phenomena occurring in normal sympathetic neurons during their development (Nakagawara, 2001). Likewise, failure to activate apoptotic programmes in response to the exposure to cytotoxic drugs may contribute to the resistance of NT cells to anticancer treatment (Fulda and Debatin, 2003).

Apoptosis is a form of programmed cell death involved in embryonic development and normal tissue omeostasis. The process can be triggered through two major pathways: (i) extracellular signals such as members of the tumour necrosis factor family can activate the death receptor-mediated extrinsic route; or alternatively (ii) stress signals such as DNA damage, hypoxia and loss of survival signals can activate the mitochondria-dependent intrinsic route (Evan and Littlewood, 1998). Both pathways involve the activation of a series of enzymes called caspases, a family of proteases that cleave after aspartic acid residues, but while each pathway has an independent group of ‘initiating’ caspases, both converge to utilise the same group of ‘effector’ caspases that execute the final cell death programme (Thornberry and Lazebnik, 1998). As a consequence, apoptotic cells, characterised at the end-stage by DNA endonuclease cleavage yielding 180 base pair fragments, acquire typical morphologic aspects such as condensed and fragmented nuclear material, usually accompanied by condensed eosinophilic cytoplasm (Majno and Joris, 1995).

In NB, multiple defects in mediators of apoptosis have been described and, among them, the increased expression of antiapoptotic molecules such as Bcl-2 (Castle *et al*, 1993) and the decrease sensitivity to tumour necrosis factor-related apoptosis-inducing ligand (TRAIL)-induced apoptosis secondary to the inactivation of caspase 8 (Hopkins-Donaldson *et al*, 2000; Teitz *et al*, 2000) appear to be the most consistent findings in aggressive tumours. The relative contribution of each of these numerous defects to spontaneous tumour regression or progression, however, remains incompletely understood.

A further critical molecule in the modulation of apoptosis in response to a series of stresses including DNA damage, hypoxia or proliferative signals is p53, whose stabilization leads cells to undergo either a cell cycle arrest or apoptosis (Prives and Hall, 1999). Cells harbouring an impaired p53 can survive and proliferate inappropriately and this event can result in cancer development (Prives and Hall, 1999). In NB, a tumour that usually shows an initial response to chemotherapy but tends to relapse as drug-resistant disease, the inactivation of p53 via mutational or nonmutational mechanisms could eventually represent one of the most critical factors of therapeutic failure (Tweddle *et al*, 2003).

Recently, gene expression profiling of human tumours has become available and several hundreds of genes differentially expressed in favourable and unfavourable NB subsets have been identified (Alaminos *et al*, 2003; Ohira *et al*, 2003). As it is unlikely that hundreds of specific and coordinated molecular alterations are required to transform a neural crest-derived progenitor cell to tumour cell, further studies could potentially identify the few seminal genes – maybe including apoptotic genes – that play a key role in the disruption of normal gene transcription resulting in the peripheral NT phenotype.

In the present study, we investigated the significance of apoptotic processes in a series of newly diagnosed peripheral NTs. Our results confirm that high MKI is associated with unfavourable clinical and biological features, but does not correlate with any specific apoptotic gene profile. Neither apoptosis nor proliferation is *per se* indicative of clinicobiological behaviour and outcome.

## MATERIALS AND METHODS

### Patients

Primary tumour samples were obtained by surgery from 110 children with newly diagnosed NT presenting between January 1985 and June 2002 to the Department of Pediatrics at La Sapienza University, the Division of Oncology at Bambino Gesù Children's Hospital and the Division of Oncology at Royal Liverpool Children's NHS Trust Alder Hey. No selection criteria were applied except for the availability of adequate frozen tumour tissue for immunohistochemical and molecular analyses.

Patients, 60 males and 50 females, were aged from 1 day to 192 months (median, 20.5). Primary site was adrenal in 57 patients, abdominal nonadrenal in 30, thoracic in 21, and cervical in two. The 110 patients were staged according to the International Neuroblastoma Staging System (INSS) (Brodeur *et al*, 1993): 21 patients were at stage 1, 23 at stage 2, 17 at stage 3, 34 at stage 4, and 15 at stage 4S.

As of June 2005, the median follow-up for the 110 patients was 88.5 months (range: 1 day to 234 months). In 66 patients either disease-free (DF, *n*=64) or alive with disease (AWD, *n*=2) at that time, a median follow-up of 124.5 months (range: 45–234 months) was observed. In 44 patients either dead of disease (DOD, *n*=42) or dead of complications (DOC, *n*=2), a median follow-up of 16 months (range: 1 day to 120 months) was recorded.

Tumours were classified according to the International Neuroblastoma Pathology Classification (INPC) (Shimada *et al*, 1999a) as follows: 90 NBs and 20 GNBs. The MKI, defined as the number of tumour cells in mitosis or karyorrhexis (morphologically recognizable by ruffling, blebbing and condensation of nuclear cromatin) out of 5000 tumour cells, was determined according to the INPC and categorized as low (<2%, equivalent to <100/5000) or high (⩾2%, equivalent to ⩾100/5000).

Institutional written informed consent was obtained from the patient's parents or legal guardians. The study underwent ethical review and approval according to local institutional guidelines.

### Tumour sample handling

At diagnosis, part of the tumour sample was formalin-fixed and paraffin-embedded for histological examination; a part was snap frozen in liquid nitrogen and stored at −80°C until Southern blot for the determination of MYCN copy number was performed as each patient was diagnosed (Dominici *et al*, 1989). Frozen tumour samples were also used to prepare the cryostat sections for the immunohistochemical analysis and to extract RNA for apoptotic gene array analysis.

### Immunohistochemistry

Frozen sections, 4 *μ*m, from the 110 tumours were acetone-fixed and then incubated with a MoAb directed against Bcl-2 (Dako, Glostrup, Denmark), and rabbit polyclonal antibodies against Bcl-X_L_, Bax and Mcl-1 (generous gift from John C Reed, The Burnham Institute, La Jolla, CA, USA). Indirect avidin-biotin immunoperoxidase staining was performed (Vectastain, Vector Laboratories, Burligame, CA, USA). The reaction product was revealed by 0.2% hydrogen peroxide and 0.6% 3,3-diaminobenzidine (Sigma, Saint Louis, MI, USA). Slides were counterstained with Mayer's haematoxylin. Cases were categorized as positive when >25% of tumour cells were immunostained. Negative controls were incubated with isotype matched nonimmune immunoglobulins and yielded no staining.

Paraffin sections, 5 *μ*m, from 60 out of 110 representative tumours selected according to MKI value, 30 with high and 30 with low MKI, were deparaffinized, rehydrated and incubated with anti-human p53 (Dako) and parkin (Cell Signaling Technology, Beverly, MA, USA), and with MoAb Ki67 (Novocastra Laboratories, Newcastle upon Tyne, UK). The antibodies were used at 1 : 50/1 : 100 final dilutions. The antigen retrieval technique was employed for the detection of all proteins. After repeated washings with PBS, sections were incubated with avidin-biotin complex kit (ABC-peroxidase, Dako). The reaction product was revealed by 0.2% hydrogen peroxide and 0.6% 3,3-diaminobenzidine (Sigma). Slides were counterstained with Mayer's haematoxylin. Negative controls were incubated with isotype matched nonimmune immunoglobulins and yielded no staining. As positive controls for parkin protein, paraffin sections from substantia nigra samples obtained from human autoptic brain specimens were utilized. Ki67 labelling index was determined by counting the number of Ki67-positive cells out of 5000 tumour cells and was expressed as the percentage positivity of each tumour for Ki67 immunostaining.

### Microarray analysis

Frozen sections, 10 *μ*m, from 40 out of 110 tumours selected according to MKI value, 20 with high and 20 with low MKI, were collected in test tubes. Total RNA was prepared using the RNA FAST KIT (Molecular System, San Diego, CA, USA) and its integrity was assessed by denaturating agarose gel electrophoresis and spectrophotometry. Microarray analysis was performed as described in detail in http://www.superarray.com. Briefly, total RNA (3 *μ*g) was used as the template for biotin-labelled cDNA probe synthesis and hybridised to APOPTOSIS oligonucleotide arrays (GEArray Q Series Human Apoptosis Gene Array, SuperArray Bioscience Corporation, Frederick, MD, USA), designed to profile the expression of 96 key genes involved in apoptosis. Each gene was tested in quadruplicate and the reaction was revealed by chemiluminescence; the image was converted into a raw data file using an image analysis software. Each array was spotted with a negative control of pUC18, blank and housekeeping genes (*β*-actin, GAPDH, cyclophilin A and ribosomal protein L13a). The relative amount of a particular transcript was estimated by directly comparing its signal intensity to the signal derived from GAPDH. To make the data comparable, each signal was normalised against the signal of housekeeping genes.

### Statistical analysis

Associations between MKI and clinical, histological and molecular features as well as between Bcl-2 family protein expression and clinical, histological and molecular features were analysed using 2 × 2 contingency tables analysis and two tails Fisher's Exact test or *χ*^2^ test. Comparisons of Ki67 labelling index between subgroups of tumours with different MKI and MYCN status were carried out using one-way analysis of variance followed by Bonferroni multiple comparisons post hoc test. Univariate and multivariate regression analysis according to the Cox proportional hazard model (Cox, 1972) and Kaplan and Meier survival curves calculation (Kaplan and Meier, 1958) were carried out using the software package SPSS 7.0 for Windows (SPSS Inc, Chicago, IL, USA).

## RESULTS

**Figure 1:**
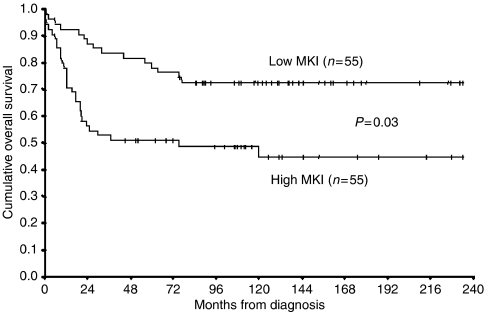
Kaplan–Meier survival analysis according to low or high MKI in 110 patients with NT. Overall survival was 73% in those with low MKI compared to 45% in those with high MKI (*P*=0.03).

In total, 110 NTs were histologically grouped in two subsets according to MKI determined following the INPC criteria (Shimada *et al*, 1999a): low MKI when mitotic and karyorrhectic cells were <2%, and high MKI when these cells were ⩾2%. Out of 110 NTs, 55 (50%) had a high MKI and 55 (50%) a low MKI. The distribution of MKI according to age at diagnosis (⩽1 year *vs* >1 year, 42 *vs* 68), primary site (nonadrenal *vs* adrenal, 53 *vs* 57), clinical stage (1, 2, 4S *vs* 3 and 4, 59 *vs* 51), INPC histological category (GNB *vs* NB, 20 *vs* 90), MYCN status (single copy *vs* amplified, 94 *vs* 16) and outcome (DF and AWD *vs* DOD and DOC, 66 *vs* 44) was studied (Table 1). High MKI was significantly associated with advanced stage (*P*=0.007), NB category (*P*=0.024), MYCN amplification (*P*<0.001), and poor outcome (*P*=0.011). When Cox simple regression analysis was applied, age at diagnosis over 1 year (*P*=0.03), adrenal primary site (*P*=0.003), advanced stage (*P*<0.001), NB category (*P*=0.01), high MKI (*P*=0.03), and MYCN amplification (*P*<0.001) were all significantly associated with a shorter overall survival (Table 2). In particular, overall survival probability was 45% in patients with high MKI compared to 73% in patients with low MKI (Figure 1). However, when Cox multiple regression analysis was applied, only advanced stage and MYCN amplification (*P*<0.001 for both variables) were independently associated with a shorter overall survival (Table 2).

We also investigated the expression of four members of the Bcl-2 family (Bcl-2, Bcl-X_L_, Bax and Mcl-1) by immunohistochemistry (IHC) on frozen sections obtained from the same 110 NTs. Immunostaining for Bax and Bcl-X_L_ was detected in the cytoplasm of undifferentiated or poorly differentiated neuroblasts, whereas positivity for Bcl-2 and Mcl-1 was detected in undifferentiated or poorly differentiated neuroblasts and in mature ganglion cells. Possible correlations between the expression of these proteins and clinical, histological and prognostic features were also investigated, but no significant associations were found except for Bcl-X_L_ to be expressed less frequently in cases over 1 year of age at diagnosis (*P*=0.021) (Table 3).

Nuclear localisation of p53 is essential for its normal function. In NB, p53 although rarely mutated (Vogan *et al*, 1993), can still be functionally inactivated through cytoplasmic sequestration secondary to multiple mechanisms (Moll *et al*, 1995; Tweddle *et al*, 2003) and, among them, even to a possible binding to parkin protein (Nikolaev *et al*, 2003). In order to determine the relevance of this phenomenon, we investigated the expression of p53 and parkin in paraffin sections from 60 out of 110 NTs, 30 with low and 30 with high MKI. Parkin was consistently negative in all cases, whereas p53 protein was observed in 47 out of 60 tumours (78%). In all cases, immunostaining for p53 was detected at nuclear level in approximately 25–50% of neuroblastic cells. Cytoplasmic staining indicative of p53 sequestration was not detectable in this series.

In order to extensively explore the expression of the genes involved in apoptosis by using a technique more sensitive than IHC, a microarray analysis designed to profile the expression of 87 key genes involved in apoptosis was performed in 40 out of 110 NTs, 20 with low and 20 with high MKI (Table 4). Four out of the 20 high MKI tumours were MYCN amplified. The results are reported as median value for each transcript. The total number of genes expressed in the low and high MKI subsets was comparable, being 63 out of 87 and 66 out of 87, respectively, the mean number of expressed genes. In total, 38 representative genes were selected and grouped as belonging to the intrinsic route (*n*=8), extrinsic route (*n*=16) or to caspases (*n*=14). Among the genes involved in the intrinsic route, those expressed at the highest level were Bax and Bcl-2L2 (bcl-w gene) in both low and high MKI tumours, immediately followed by survivin and Mcl-1. Among the genes involved in the extrinsic route, TRAIL-R1 (DR4) and TRAIL-R2 (DR5) were expressed at the highest level in both low and high MKI tumours, followed by TRAF-1, TRAF-6, Fas and Fas-ligand. TRAIL was poorly expressed in both groups, even though the amount of its mRNA was double in tumours with high MKI. In low and high MKI tumours, p53 gene was equally expressed at very low levels. Among the caspases, caspase 5, 7 and 14 were the genes expressed at the highest level. In particular, caspase 7 and 14 were the genes expressed at the highest level in low MKI tumours, whereas caspase 7 and 5 those expressed at the highest level in high MKI tumours. Only low levels of caspase 8 mRNA were documented in both subsets. The levels of expression of the 38 selected genes were statistically analysed, but no obvious correlation was shown with histological, clinical and prognostic features.

**Figure 2:**
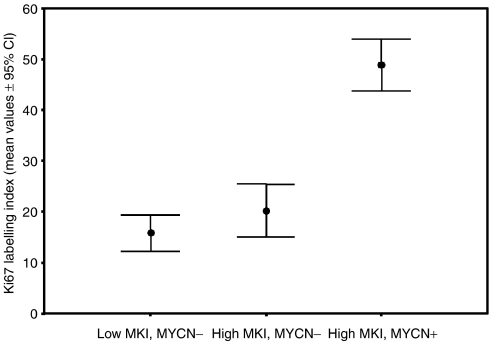
Distribution of Ki67 labelling index (mean values±95% confidence interval) between subgroups of tumours with different MKI (low *vs* high) and MYCN status (single copy *vs* amplified). Abbreviations – MYCN−: MYCN single copy; MYCN+: MYCN amplified; CI: confidence interval.

Owing to the difficulty in distinguishing mitotic cells from other karyorrhectic tumour cells, in the Shimada system they are counted together to give the MKI. Therefore, in order to evaluate the relevance of the mitotic component in this series of NTs, proliferative activity was investigated using Ki67 immunostaining in paraffin sections from 60 NTs, 30 with low MKI (all MYCN single copy) and 30 with high MKI (23 single copy and seven MYCN amplified). Our results showed that the mean value of Ki67 labelling index (as defined in Materials and Methods) was 15.9±9.6 in the low MKI subset and 26.9±16.3 in the high MKI subset (*P*=NS). However, when the Ki67 labelling index mean value of the seven MYCN amplified NTs was calculated (48.9±5.5), it was found to be significantly higher than in the 23 high MKI (20.3±12.0) and the 30 low MKI tumours (*P*<0.001) (Figure 2). Among the 53 MYCN single copy tumours, no significant difference in Ki67 labelling index was detected between those with low or high MKI.

## DISCUSSION

In the present study, we investigated the relevance of apoptosis in peripheral NTs and the extent to which it contributes to determine the clinical significance of MKI. Our results show that a high MKI is associated with advanced stage, NB histological category, MYCN amplification and poor outcome, thus confirming previous findings reported by Shimada *et al* (1995, 1999b, 2001) and Ambros *et al* (2002). However, when the levels of expression of key genes involved in apoptosis were compared to either MKI and clinicobiological features, no significant associations were found.

Multiple defects in intrinsic and extrinsic routes to apoptosis and in caspases have been described in NTs. In the intrinsic route, increased levels of the antiapoptotic protein Bcl-2 in primary NBs correlate with unfavourable histology according to Shimada and with MYCN amplification (Castle *et al*, 1993), and seem to promote tumour cell survival (Ikeda *et al*, 1995). These findings, however, were not confirmed by other reports in which no association with MYCN amplification was found (Ikegaki *et al*, 1995). Similarly, overexpression of Bcl-2 and Bcl-X_L_ in NB cell lines was described to block chemotherapy-induced apoptosis, thus suggesting that the expression of these proteins may promote the drug resistance characteristic of high-risk NBs (Dole *et al*, 1994, 1995). In the present study, however, no significant associations between the profile of genes involved in the intrinsic route to apoptosis and histological, clinical and prognostic features of NTs were found. In particular, microarray and immunohistochemical analysis demonstrated that the expression of the antiapoptotic proteins Bcl-2, Bcl-X_L_ and Mcl-1 was not associated with either MYCN amplification or other unfavourable prognostic features. However, taking into account that survival and death signals converge on the mitochondria through proapoptotic (Bax, Bak, Bid, Bad, Bik, etc.) and antiapoptotic (Bcl-2, Bcl-X_L_, Mcl-1, A1 and Bcl-w) proteins and that the former promote mitochondrial cytochrome *c* efflux into cytosol while the latter inhibit the same efflux (Cory and Adams, 2002), it is expected that the critical determinant of cell survival is the overall balance between the pro- and antiapoptotic members at mitochondrial level rather than the concentration of the individual members.

In the extrinsic route, TRAIL selectively promotes apoptosis in most primary tumours and tumour cell lines, and this process can be potentiated by cytotoxic agents (Ashkenazi and Dixit, 1999). Two agonistic receptors for TRAIL, TRAIL-R1 (DR4) and TRAIL-R2 (DR5, KILLER), and two antagonistic receptors, TRAIL-R3 (DcR1) and TRAIL-R4 (DcR2), have been identified (Ashkenazi and Dixit, 1999). Activation of TRAIL-R1 and TRAIL-R2 activates a signal transduction cascade involving several molecules and, among them, caspase 8 and 10 (Özören and El-Deiry, 2003). In NB, resistance to TRAIL-induced apoptosis has been preferentially described in MYCN-amplified tumours and correlates with inactivation of caspase 8 gene secondary to gene deletion or silencing through DNA hypermethylation (Hopkins-Donaldson *et al*, 2000; Teitz *et al*, 2000). Sensitivity to TRAIL-induced apoptosis can be restored in caspase 8- and 10-deficient NB cell lines by inducing a stable re-expression of caspase 8 (Muhlethaler-Mottet *et al*, 2003). In the present study, among the genes involved in the extrinsic route, TRAIL-R1 (DR4) and TRAIL-R2 (DR5) were expressed at high level in both favourable and unfavourable NTs, and no associations with MYCN amplification or other prognostic features were shown. In agreement with previous studies reporting a loss of caspase 8 expression in aggressive NBs (Hopkins-Donaldson *et al*, 2000; Teitz *et al*, 2000; Stupack *et al*, 2006), in our series a low expression of caspase 8 mRNA was found in all tumours, with a tendency for high risk tumours to express even lower levels.

TRAIL-R3 and TRAIL-R4, the two decoy receptors unable to transduce the death signal, are thought to block TRAIL-induced apoptosis by competing with TRAIL-R1 and TRAIL-R2 for the ligand (Ashkenazi and Dixit, 1999; Özören and El-Deiry, 2003). Hence, cells that express TRAIL-R3 and/or TRAIL-R4 at high levels compared to TRAIL-R1 and/or TRAIL-R2 may use the decoys as protection against TRAIL-induced apoptosis, although this is not the only mechanism of resistance to TRAIL (Ashkenazi and Dixit, 1999; Özören and El-Deiry, 2003). Downregulation of TRAIL-R3 and TRAIL-R4 expression through hypermethylation of their promoters has been previously described in NB cell lines (van Noesel *et al*, 2002). The present findings, showing that TRAIL-R3 and TRAIL-R4 were expressed at substantially lower levels than TRAIL-R1 and TRAIL-R2, are in agreement with this report and seem to exclude that this mechanism of resistance to TRAIL is active in NB tumours.

TRAIL-R2 has been identified as a transcriptional target of p53 (Wu *et al*, 1997). In NB, p53 is rarely mutated (Vogan *et al*, 1993) but can be functionally inactivated through cytoplasmic sequestration (Moll *et al*, 1995). Several mechanisms have been proposed (Tweddle *et al*, 2003) and, among them, a possible binding to parkin protein (Nikolaev *et al*, 2003). Regardless of the mechanism – mutational or nonmutational – underlying p53 inactivation, this could eventually represent one of the most critical factors of therapeutic failure in NB tumours (Tweddle *et al*, 2003). Accordingly, in the present study, the levels of p53 mRNA expression were very low in both favourable and unfavourable tumours, with a tendency for high-risk tumours to express even lower levels. Moreover, when immunohistochemical analysis was carried out, immunostaining for p53 was only detected in the nucleus of neuroblasts and parkin protein was never detected. These findings suggest that p53 cytoplasmic sequestration is an infrequent event in NB tumours and likely unrelated to the binding to parkin protein.

In the Shimada system, due to the difficulty in distinguishing morphologically mitotic from karyorrhectic tumour cells, these are counted together to give the MKI. In this study, the absence of correlations between apoptotic features and MKI prompted us to investigate whether and to what extent tumour cell proliferative activity may also account for the different clinicobiological behaviours. No significant differences in Ki67 labelling index were demonstrated in the low *vs* high MKI subset of NTs, the only difference being confined to the MYCN amplified tumours. A previous report suggested that, since proliferating cells generally outnumbers apoptotic cells, the resulting sum relies most heavily on proliferation and this is the reason why a high MKI correlates with a poor outcome in NTs (Gestblom *et al*, 1995). This study confirmed that proliferating cells outnumber apoptotic cells but also showed that proliferating cells are equally present in low and high MKI tumours. A significantly higher proliferative activity was only detected in the small subgroup of MYCN amplified tumours.

In conclusion, the present findings confirm previous reports indicating that MKI is in NTs a reliable morphological marker of clinical behaviour and identifies favourable and unfavourable NTs (Shimada *et al*, 1995, 1999b, 2001; Ambros *et al*, 2002). Our results also show that karyorrhectic cells represent the end-stage of a cascade of events leading to morphologically recognisable apoptosis, although none of these individual events is able by itself to determine the clinical relevance of the entire apoptotic process. Neither proliferation nor apoptosis is *per se* indicative of clinicobiological behaviour and outcome. In NTs, prognostication still remains strongly dependent on the morphological assessment of MKI.

## Figures and Tables

**Table 1:** Distribution of MKI according to clinical, histological and prognostic features in 110 NTs

**Features**	**No. of cases**	**Low MKI**	**(%)**	**High MKI**	**(%)**	***P* value**
*Age at diagnosis*						
⩽1 year	42	23	(55)	19	(45)	NS
>1 year	68	32	(47)	36	(53)	
						
*Primary site*						
Nonadrenal	53	32	(60)	21	(40)	NS
Adrenal	57	23	(40)	34	(60)	
						
*Clinical stage*						
1, 2, 4S	59	37	(63)	22	(37)	0.007
3, 4	51	18	(35)	33	(65)	
						
*INPC category*						
GNB	20	15	(75)	5	(25)	0.024
NB	90	40	(44)	50	(56)	
						
*MYCN status*						
Single copy	94	54	(57)	40	(43)	<0.001
Amplified	16	1	(6)	15	(94)	
						
*Outcome*						
DF, AWD	66	40	(61)	26	(39)	0.011
DOD, DOC	44	15	(34)	29	(66)	

Abbreviations: AWD: alive with disease; DF: disease free; DOD: dead of disease; DOC: dead of complication; NS: nonsignificant.

**Table 2:** Simple and multiple Cox regression analysis of the correlations between overall survival and clinical, histological and prognostic features in 110 NTs

	**Cox simple regression analysis**				**Cox multiple regression analysis**		
**Features**	**OS%**	**RR**	**(CI)**	***P***	**RR**	**(CI)**	***P***
*Age at diagnosis*							
⩽1 year	76	2.33	(1.15–4.72)	0.02	1.70	(0.77–3.77)	NS
>1 year	48						
							
*Primary site*							
Nonadrenal	75	2.65	(1.39–5.08)	0.003	1.63	(0.83–3.11)	NS
Adrenal	45						
							
*Clinical stage*							
1, 2, 4S	92	14.46	(5.65–36.97)	<0.001	7.01	(2.46–19.94)	<0.001
3, 4	21						
							
*INPC category*							
GNB	86	6.28	(1.52–25.97)	0.01	3.01	(0.70–12.82)	NS
NB	53						
							
*MKI*							
Low	73	2.61	(1.39–4.88)	0.03	1.19	(0.59–2.42)	NS
High	45						
							
*MYCN status*							
Single copy	69	12.94	(6.18–27.12)	<0.001	5.64	(2.42–13.13)	<0.001
Amplified	0						

Abbreviations: CI: 95% confidence interval; P: statistical probability; NS: statistically not significant; OS%: percentage of overall survival; RR: relative risk.

**Table 3:** Distribution of immunostaining for four members of Bcl-2 family according to clinical, histological and prognostic features in 110 NTs

**Features**	**Bcl-2**	**(%)**	***P* value**	**Bcl-X_L_**	**(%)**	***P* value**	**Bax**	**(%)**	***P* value**	**Mcl-1**	**(%)**	***P* value**
No. of +ve cases/No. of total cases	80/110	(73)	NA	64/110	(58)	NA	64/110	(58)	NA	55/110	(50)	NA
												
*Age at diagnosis*												
⩽1 year (*n*=42)	31	(74)	NS	30	(71)	0.021	26	(62)	NS	23	(55)	NS
>1 year (*n*=68)	49	(72)		34	(50)		38	(56)		32	(47)	
												
*Primary site*												
Non adrenal (*n*=53)	41	(77)	NS	32	(60)	NS	31	(59)	NS	25	(47)	NS
Adrenal (*n*=57)	39	(68)		32	(56)		33	(58)		30	(53)	
												
*Clinical stage*												
1, 2, 4S (*n*=59)	41	(70)	NS	37	(63)	NS	33	(56)	NS	28	(48)	NS
3, 4 (*n*=51)	39	(77)		27	(53)		31	(61)		27	(53)	
												
*INPC category*												
GNB (*n*=20)	14	(70)	NS	10	(50)	NS	11	(55)	NS	13	(65)	NS
NB (*n*=90)	66	(73)		54	(60)		53	(59)		42	(47)	
												
*MKI*												
Low (*n*=55)	41	(75)	NS	32	(58)	NS	29	(53)	NS	28	(51)	NS
High (*n*=55)	39	(71)		32	(58)		35	(64)		27	(49)	
												
*MYCN status*												
Single copy (*n*=94)	69	(73)	NS	54	(57)	NS	53	(56)	NS	45	(48)	NS
Amplified (*n*=16)	11	(69)		10	(63)		11	(69)		10	(63)	
												
*Outcome*												
DF, AWD (*n*=66)	51	(77)	NS	42	(66)	NS	38	(58)	NS	36	(55)	NS
DOD, DOC (*n*=44)	29	(67)		22	(50)		26	(59)		19	(43)	

Abbreviations: AWD: alive with disease; DF: disease free; DOD: dead of disease; DOC: dead of complication; NA: nonapplicable; NS: statistically not significant.

**Table 4:** Apoptotic gene profile in 20 NTs with low MKI and 20 NTs with high MKI: genes are grouped as belonging to the intrinsic route (*n*=8), extrinsic route (*n*=16) or to caspases (*n*=14)

	**Low MKI**	**High MKI**
Expressed genes/total no. of genes (range)	63/87 (26–87)	66/87 (34–86)
Genes	Median	Median
		
*Intrinsic route*		
BAX	33352	30476
Bcl-2	5807	5043
BCL2A1 (bfl-1 gene)	1585	3159
BCL2L11 (bimL gene)	3043	2943
BCL2L2 (bcl-w)	26702	17443
Bcl-x	3367	4004
Mcl-1	14412	6726
Survivin	10675	15771
		
*Extrinsic route*		
Fas	8287	8422
Fas ligand	6267	8218
FADD	1162	2387
TRAF1	11939	8068
TRAF2	1171	878
TRAF3	1560	1615
TRAF4	710	1196
TRAF5	1015	760
TRAF6	7602	8370
TRAIL	2181	4740
TRAIL-R1 (DR4)	15603	9182
TRAIL-R2 (DR5, KILLER)	37873	24555
TRAIL-R3 (DcR1)	4534	6042
TRAIL-R4 (DcR2)	1755	2392
TNF*α*	6183	3464
p53	1384	570
		
*Caspases*		
CASP1	8026	8324
CASP2	3178	2559
CASP3	3133	5544
CASP4	2319	9125
CASP5	8891	18756
CASP6	3980	7378
CASP7	23764	14893
CASP8	5143	2663
CASP8AP	6881	3878
CASP9	445	1604
CASP10	4881	4154
CASP13	3572	3236
CASP14	20611	8726
CASPER	2264	3741
